# Editorial Comment to “Management of apalutamide‐induced rash with focus on early peaks”

**DOI:** 10.1111/iju.15611

**Published:** 2024-10-22

**Authors:** Sayuri Takahashi

**Affiliations:** ^1^ Department of Urology The Institute of Medical Science, the University of Tokyo Tokyo Japan

Apalutamide is one of the androgen receptor signaling inhibitor (ARSi) agents, and often prescribed for the patients diagnosed as high‐risk prostate cancer or castration‐resistant prostate cancer with androgen deprivation therapy (ADT). Urologists often see patients who develop skin rash during the apalutamide treatment. Incidences of skin rash were frequent in a phase 3 study, with 23.8–29.2% of patients, and was distinctly high in Japanese patients, with rates of 32.3–53.6%.^1^ Alexander Pan et al. reported apalutamide‐related dermatologic adverse events (dAEs) are frequent and can be managed with topical ± oral steroids.^2^ With expanded approval of apalutamide, dAE identification and management are essential.^2^ Alison et al. recently stated the importance of preventing skin problems and managing them at the earliest state.^3^ They developed practical guidance for the management of apalutamide‐related dAEs by grade.^3^ The early identification of rash and subsequent intervention resulted in quicker resolution and continuation of the cancer treatment.^3^

The present study by Hashimoto et al. was performed to determine the effect of early interventional protocol (EIP) developed by them on the continuation of apalutamide treatment.^1^ In this EIP, a moisturizing lotion, oral antihistamines, fexofenadine 60 mg twice daily, and topical corticosteroid, clobetasol propionate ointment were prescribed at the start of apalutamide treatment.^1^ Then, patients started to use the medicine when rashes appeared by their own assessment with a reduced dose of apalutamide. Patients were also explained to capture a photograph of the rash and contact the clinic. In case, patients had fever, pain, or rashes with erosions or blisters, they were instructed to discontinue the apalutamide and consult a dermatologist.

In this study, 37 patients treated with apalutamide were enrolled and 15 patients had skin rash (40.5%). Apalutamide was continued at a reduced dose in 12 patients and discontinued in 2 patients during rash treatment. Only one patient terminated apalutamide treatment and systemic corticosteroids were administered by a dermatologist.

The incidence rate was similar with previous studies, however, this study showed EIP could elevate the rate of apalutamide continuation at a reduced dose with skin‐rash treatment. There may exist an issue of the insurance medical treatment when topical corticosteroid, clobetasol propionate ointment which are strong medicine are prescribed for patients without any symptoms. At busy outpatient clinics with a full of patients, it is sometimes hard to care patients when skin rashes appeared expeditiously. Therefore, the education to patients and prescription in advance by following this EIP seems to be reasonable and useful for both patients and urologists.

The EIP would be one of helpful methods on the treatment of castration‐resistant prostate cancer.

## CONFLICT OF INTEREST STATEMENT

None declared.
